# Fabrication and analysis of a PVP-carboxymethyl chitosan/forsterite nanocomposite scaffold with stainless steel base via freeze-drying and neural network techniques

**DOI:** 10.22038/ijbms.2025.89859.19382

**Published:** 2026

**Authors:** Negin Ghanbari, Bahareh Kamyab Moghadas, Fereshteh Samadi, Amirsalar Khandan

**Affiliations:** 1 Department of Chemical Engineering, Shi.C., Islamic Azad University, Shiraz, Iran; 2 Dental Research Center, Dental Research Institute, School of Dentistry, Isfahan University of Medical Sciences, Isfahan, Iran

**Keywords:** 3D printer, Artificial neural network, Bone scaffold, Forsterite, Nanoparticles

## Abstract

**Objective(s):**

This study aims to design and fabricate innovative polymer-ceramic-metal scaffolds for bone tissue engineering, utilizing 3D printing and freeze-drying techniques to enhance bone repair.

**Materials and Methods:**

Stainless steel scaffolds were produced via selective laser melting (SLM) and coated with varying weight percentages (0, 5, 10, 15) of polyvinylpyrrolidone (PVP), carboxymethyl chitosan (CMC), and forsterite using freeze-drying. The scaffolds were characterized through Fourier transform infrared spectroscopy (FTIR), X-ray diffraction (XRD), and scanning electron microscopy (SEM) to assess functional groups, phase purity, porosity, and pore size. Biological assessments included bioactivity, ion emission tests (ICP-AES), and wettability evaluations. Artificial neural networks (ANN) were employed to predict mechanical and biological properties.

**Results:**

The analysis revealed that scaffolds with 15% forsterite exhibited optimal mechanical and biological performance, enhancing the scaffold’s potential for clinical applications in bone repair.

**Conclusion:**

This study introduces a novel scaffold design that significantly improves bone tissue regeneration processes. The integration of advanced materials and predictive modeling through ANN paves the way for future research in the field of bone tissue engineering.

## Introduction

Bone is a hard and stable organ in the human and animal body that acts as the body’s framework and as an internal protector of vital organs. Bones are a combination of various minerals, such as calcium and phosphorus, in the form of fibers and living cells that are located within a hard matrix and form a hard structure of bone. Bones are the site for the manufacture of white and red blood cells, provide movement in joints, and maintain the body’s surface for balance and support (1-3). Bone is a unique tissue that is physiologically constantly being destroyed and rebuilt. Bone remodeling is a continuous process of bone resorption and formation carried out by different types of bone cells. This process continues throughout life, but remains in positive balance only until the age of 30. The process of bone remodeling begins with the activation of bone remodeling units (4, 5). 

The bone remodeling process is composed of bone cells, including osteoclasts and osteoblasts, which work together to remodel bone tissue. Osteoblasts and osteoclasts have distinct functions and originate from different developmental lineages, and both cell types undergo a short life cycle consisting of activation, activity, and final elimination (after their work is complete, they undergo apoptosis (programmed cell death)) (6, 7). Therefore, bone tissue naturally has a regenerative capacity, and the body uses this ability to deform or repair bone following injuries such as bone fractures or minor injuries that occur during normal daily activities. However, sometimes bone injuries are very extensive (usually more than 2 cm, depending on the anatomical location), and are such that spontaneous repair is not possible. As a result, clinical intervention is required to achieve functional restoration and complete recovery. Therefore, the field of bone tissue engineering for the repair and regeneration of bone lesions has witnessed significant advances in the field of biomaterials, which have facilitated the process of bone regeneration at the site of the defect without incurring any risks. 

 The goal of bone tissue engineering is to design materials that perform better than autografts and allografts. These materials can be inserted into bone defects and temporarily act as an extracellular matrix in the tissue. These materials are often prepared in the form of scaffolds and act as a support structure for cell attachment (8-10). Tissue engineering approaches have been considered for the repair and regeneration of bone lesions. The role of an ideal scaffold that helps cell adhesion and growth is of particular importance (11, 12). An ideal scaffold for bone tissue engineering applications should provide or improve cell viability, attachment, proliferation, osteogenic differentiation, vascularization, integration with host tissue, and, if necessary, load-bearing (13-15). The characteristics of tissue engineering scaffolds can be divided into four main categories, including biological characteristics, structural properties, biomaterial composition, and types of manufacturing processes. Most scaffolds currently used for bone tissue engineering applications are composed of polymers, bioactive ceramics (Bioactive Glasses), and composites (16-18). Several materials used to fabricate bone scaffolds include metals (such as titanium alloys), bio ceramics (such as zirconia and alumina), and synthetic polymers (such as polyetheretherketone and polymethyl methacrylate). In bone tissue engineering, biomedical materials (biomaterials) serve as the main components of scaffolds (19, 20). The goal of biomaterials is to evaluate, treat, enhance, repair, or replace tissues or organs in the body. An optimal biomaterial should be bone-conductive in vivo, biodegradable, non-cytotoxic, printable, and bioactive (21). 

Given the very important role of scaffolds in tissue engineering, there are various methods for manufacturing and designing bone scaffolds, and by choosing the appropriate manufacturing method, a better contact surface is provided for cell attachment, proliferation, and differentiation. Existing technologies for manufacturing 3D scaffolds can be divided into two categories: conventional and advanced. Conventional methods use subtractive methods in which portions of material are removed from an initial block to achieve the desired final scaffold. The limited ability to control shapes, complex geometries, and porosity is a disadvantage of these methods. In addition, most of these methods use organic solvents that affect cell viability. Gas foaming, solvent casting, scaffold fabrication by freeze drying, phase separation, powder-forming process, sol–gel technique, and electrospinning are examples of conventional methods. Rapid prototyping is one of the most advanced scaffold fabrication methods that, unlike conventional methods, allows for precise control over the porosity, pore size, and mechanical and chemical properties of the scaffold, allowing for better simulation of natural bone. In general, these methods do not use toxic organic solvents, which leads to increased biocompatibility of the scaffolds. Stereolithography (SL), Fused Deposition Modeling (FDM), Laser Sintering (SLS), and 3D Bioprinting (3DP) are among the most important additive manufacturing methods (22-24). 

There are various methods, including design of experiments, optimization, and artificial neural networks (ANNs), to analyze the relationships between process parameters and improve the final product performance. The design of experiments systematically plans experiments to identify important factors, and the optimization method focuses on finding the best combination of conditions. However, the design of experiments and optimization methods has limitations when dealing with complex and nonlinear relationships between parameters. In contrast, ANN can model and predict complex, multidimensional, and nonlinear relationships between parameters and scaffold properties with high accuracy. ANNs are computational models inspired by the human brain. They are used in various fields, including machine learning, to recognize patterns, make predictions, and solve complex problems. ANNs are used in image and speech recognition, natural language processing, and even in medical diagnosis. They are powerful tools for tasks involving large amounts of data and complex patterns. Therefore, in this research, the metal scaffold core was fabricated using additive manufacturing and 3D metal printing and coated with polymer and ceramic materials using freeze-drying. The aim of integrating these two methods is to prevent side effects and physical damage, enhance mechanical properties, prevent oxidation, and ensure particle uniformity. In the continuation of this project, an ANN was used to better understand the conditions and the effect of each parameter’s behavior on the others, as well as to find the relationship between the parameters. ANN is a practical method for learning various functions, such as real-valued functions, discrete-valued functions, and vector-valued functions.

## Materials and Methods

*Scaffolding preparation*

To prepare the polymer composite, after preparing a solution of 10 g of carboxymethyl chitosan in 130 ml of double-distilled water and dissolving 4 g of polyvinylpyrrolidone in 50 ml of double-distilled water, both solutions were prepared separately at a temperature of 50 °C and a stirring speed of 80 revolutions per minute (rpm). Next, the polyvinylpyrrolidone solution was slowly added to the carboxymethyl chitosan solution, and the resulting mixture was stirred until completely uniform. Then, forsterite (Fo) was added to the polymer composite at a weight percentage of (0-5-10-15), and after dissolving, it was poured into a suitable container for coating around the stainless-steel metal scaffold used by the SLM 3D printer model M100P. Finally, the metal-ceramic composite scaffold was placed in a freezer for 24 hr at a temperature of -48 °C and then for 24 hr at a temperature of -51 °C and a pressure of 0.01 millibar in a freeze-drying device.

*Material properties*

The phosphate-buffered saline (PBS) weight loss test involves measuring the change in weight of a material or sample while it is immersed in PBS. This test is used to assess biodegradation or degradation in a controlled environment. PBS is a buffer solution with a pH of 7 that is prepared by mixing sodium chloride, sodium phosphate, potassium chloride, and potassium phosphate in distilled water. In this test, the sample is immersed in PBS for 21 days. The weight of the sample is recorded before immersion and then every seven days thereafter to measure any weight change that could indicate the extent of degradation, corrosion, or dissolution of the material in the solution. The pH of the samples was also measured during this period with a pH meter. Emission spectroscopy is a chemical analysis technique that uses the intensity of light from a flame, plasma, electric arc, or spark at a specific wavelength to determine the amount of an element in a sample (25). The principle of ICP-AES involves exciting atoms in a sample solution using a high-temperature inductively coupled plasma. When the excited atoms return to their ground state, they emit light at wavelengths characteristic of each element. The intensity of the emitted light is proportional to the concentration of the element in the sample (26). In this experiment, inductively coupled plasma atomic emission spectrometry (ICP-AES) was used to determine the calcium concentration in a sample that had been in PBS solution for 21 days. After calibrating the device, the relationship between emission intensity and concentration was set. The sample solution was transferred into the system by a peristaltic pump and, after atomization, was introduced into the plasma at a temperature of 10,000 Kelvin. Under these conditions, the atoms and ions present were excited and, when they returned to the ground state, emitted light of a specific wavelength. This light was separated by the spectroscopic system, and its intensity was recorded by the detector. Finally, the calcium concentration was calculated using a calibration curve, and to ensure the accuracy of the results, control solutions and standard reference samples were also used. The wettability test is used to determine the degree of wetting or adhesion of a liquid to a solid surface. It measures the ability of a liquid to spread and form a thin film on the surface, which indicates the interaction between the liquid and the surface. In this test, a drop of the desired liquid is placed on the scaffold surface, and the contact angle formed between the liquid drop and the surface is measured. The contact angle is a key parameter that reflects the interaction between the liquid, solid, and gas phases at the interface. A contact angle of less than 90 degrees indicates favorable wetting, while a contact angle of more than 90 degrees indicates unfavorable wetting (27). Fourier transform infrared spectroscopy (FTIR) is a very versatile and valuable analytical technique used to analyze the chemical composition and molecular structure of a wide range of materials. It operates on the principle that different chemical bonds within a molecule absorb infrared radiation at specific frequencies corresponding to their vibrational modes. 

FTIR works by passing infrared light through a sample and analyzing it with different wavelengths of light. The instrument measures the intensity of the light absorbed or transmitted and produces an infrared spectrum that contains peaks corresponding to the vibrations of the molecular bonds present in the sample. By interpreting these peaks and comparing them with reference spectra, they can identify functional groups in the sample and gain insight into its molecular structure. Infrared spectra can be classified into three main regions based on the types of molecular vibrations they exhibit, the near infrared (NIR) region, wavelength range 4000–14000 , the mid-infrared (MIR) region, wavelength range 400–4000 , and the far-infrared (FIR) region (wavelength range 400–25 . The mid-infrared region is most useful for identifying functional groups and provides information about the chemical bonds present in the molecule. An X-ray diffraction (XRD) analysis is a technique used to investigate the structure of materials at the atomic or molecular level. By exposing a sample to X-rays, the diffraction patterns produced can reveal detailed information about the crystal structure, phases present, lattice parameters, and orientation of the material. 

In X-ray diffraction analysis, a monochromatic beam of X-rays is directed at a crystalline sample. When the X-rays interact with atoms in the crystal lattice, they undergo constructive interference, resulting in the X-rays being scattered in specific directions. These scattered X-rays produce a diffraction pattern that contains information about the arrangement of atoms within the crystal lattice. Bragg’s law plays a fundamental role in X-ray diffraction analysis.

ny=2dsinθ

 Equation 1

n is an integer that represents the order of the diffraction peak, λ is the spacing between atomic planes in the crystal lattice, d is the spacing between atomic planes in the crystal lattice, and θ is the angle of incidence of the X-rays. By measuring the diffraction angle (θ) and knowing the wavelength of the X-rays, researchers can use Bragg’s law to determine the interfacial spacing (d) of the crystal lattice. This information is essential for identifying the crystal structure and phase composition of the material under study. Another important concept in XRD analysis is the Scherrer relationship. The Scherrer relationship estimates the average crystallite size of a material based on the spread of diffraction peaks observed in the XRD pattern. This information is valuable for understanding the physical properties and behavior of nanomaterials and materials with microstructures.

 Equation 2

β=kλ/Dcosθ

β is the peak width at half height, κ is the Scherrer constant (usually about 0.9), D is the average crystallite size, λ is the X-ray wavelength, and θ is the diffraction angle.

Scanning electron microscopy (SEM) was used to evaluate the microstructure of the fabricated scaffold. The samples were bonded to a carbon binder and placed on a holder. They were then coated with gold (5 nm) using chemical vapor deposition (CVD) and imaged under a TESCAN-Vega3 scanning electron microscope. SEM images allow us to observe the differences between the designed pores of the scaffold and those observed after the fabrication process, with grain size changes. In addition, energy dispersive X-ray spectroscopy (EDX) in the context of structural investigation provides information about the chemical composition of different phases in a material, such as nanocomposite scaffolds. This information is crucial for understanding the chemical bonding, phase composition, and homogeneity of nanocomposite scaffolds, which are important factors affecting mechanical properties. In this study, to predict the changes in Poisson’s ratio, porosity percentage, and degeneration along nine experimental samples tested in elastic modulus (MPa) and compressive strength (MPa) with a wide range between 0,5,10,15 wt%, which is a progressive ANN, has been used. For this purpose, the neural network is formed with the inputs of elastic modulus (MPa) and compressive strength, a hidden layer with five neurons, considering the number of inputs multiplied by 2 plus one neuron for faster convergence of the results, and the outputs of Poisson’s ratio, porosity percentage and degeneration. The nonlinear sigmoid function is used for the activation function, or in other words, the hypothesis function. The reason for using the nonlinear sigmoid function is its nonlinear nature, which allows it to predict answers with higher accuracy, and the convergence of the network to the required predictions is faster. At each stage of the network progression, to train and ultimately estimate the results, the error function is optimized using the gradient descent algorithm. In addition, for higher accuracy estimation and convergence of the ANN, the input data from Table 1 is first normalized and then, after the final estimation of the results, they are denormalized so that the results are in the approved range. The fitted graph according to the linear regression method is compared with the y=x graph of the 100% accurate estimate according to the input targets from Table 1 to determine the error of the ANN. Also, to check the accuracy of the ANN in predicting the results, the network error is determined by examining it with linear regression.

**Figure 1:**
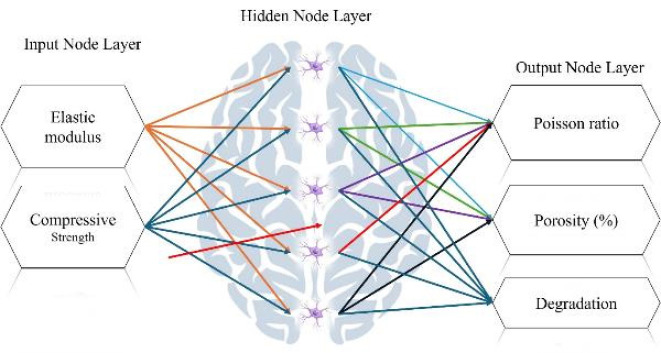
Schematic representation of an ANN with a hidden layer, fabrication of a three-component metal-ceramic-polymer bone scaffold using a metal 3D printer and freeze-drying

For this purpose, the predicted results are determined in a normalized form, and a graph is fitted to the estimated results at different points. The results obtained from the ANN formed in this study will be examined in the following. Figure 1 is a schematic of the ANN formed with a hidden layer consisting of 5 neurons and two inputs of elastic modulus (MPa) and compressive strength (MPa) in 9 samples to predict Poisson’s ratio, porosity percentage, and degeneration. ANNs are composed of interconnected nodes (neurons) organized into layers. Each neuron processes data and passes the result to the next layer. ANNs learn from data through a process called training, in which the network adjusts its weight based on the error of its predictions. This process is often done using backpropagation (28, 29).

## Results

ICP-AES analysis was used to determine the calcium ion concentration, as shown in Tables 2 and 3. First, the control sample with a value of 0.529511 ppm was taken as a reference. Then, the calcium release values in the stainless-steel samples were examined. The SS stainless steel sample had a calcium release value of 17.2089 ppm, which indicates the dissolution of calcium ions from the stainless-steel surface. In the sample SS. S1, with the addition of carboxyl methyl chitosan and PVP, the calcium content increased to 58.8468 ppm. In the sample SS. S2w with the addition of 5% Fo, the calcium content reached 21.9914 ppm, which indicates the positive effect of Fo on the release of calcium ions. In the sample SS. S3w with 10% Fo, the calcium content decreased to 87.3875 ppm, which is due to Fo. Finally, in the SS. S4w sample with 15% Fo, the calcium content increased to 239.820 ppm, which was the highest among the stainless-steel samples. Also, the calcium content was investigated in the non-stainless-steel samples S1 to S4. The addition of carboxyl methyl chitosan and PVP to the S1 sample led to an increase in the calcium content to 39.9399 ppm. By adding 5% Fo to the S2 sample, the calcium content increased to 111.064 ppm, and by increasing the percentage of Fo to 10 wt% and 15 wt% in the S3 and S4 samples, the calcium content reached 86.2108 and 338.696 ppm, respectively. Therefore, it was observed that the addition of Fo to different percentages has a significant effect on increasing the release of calcium ions (30-32). Also, samples containing 15 wt% Fo have the highest amount of calcium release, which is due to the bioactive properties and high solubility of Fo. The surface wettability or hydrophilicity of a scaffold material can significantly affect cell behavior and interactions within the scaffold. A highly hydrophilic surface promotes better cell adhesion, spreading, proliferation, and differentiation. 

**Figure 2:**
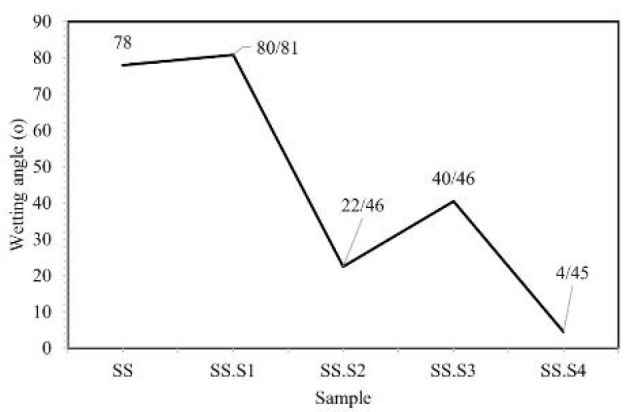
Wettability test results for sample of SS, SS.S1, SS.S2, SS.S3 and SS.S4 used in this study

**Figure 3:**
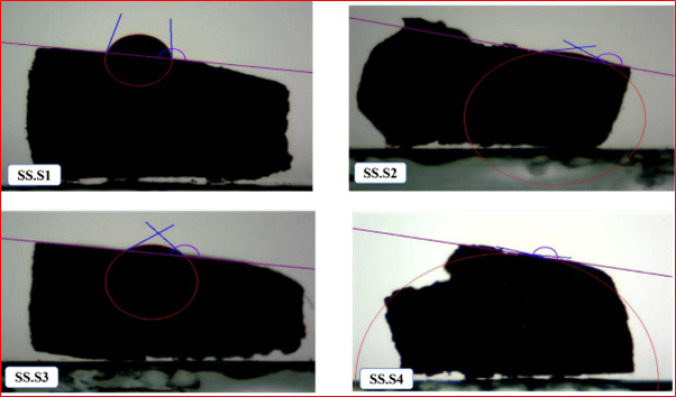
Wettability test results and image of samples at the contact angle of a drop with a diameter of 5 μm in the wettability test

On the other hand, a hydrophobic surface may inhibit these cellular processes1. As shown in Figure 2, sample SS with a contact angle of 78 indicates that the surface is relatively hydrophobic and water diffuses on it to a limited extent. Sample SS. S1 with a contact angle of 81.80 indicates the effect of carboxyl methyl chitosan and PVP in reducing the hydrophilicity of the surface. In the sample SS. S2 with the addition of 5% Fo, the sharp decrease in the contact angle indicates the improvement of the hydrophilicity of the surface because of Fo, which releases alkali ions and makes the surface more suitable for water absorption. In the sample SS. S3 with the addition of 10% Fo, the contact angle again increased and reached 40.46. This change may be due to the saturation of the surface with Fo ions and the reduction of their uniform dispersion. In the SS. S4 sample with the addition of 15 wt% Fo, the contact angle was 4.45°, a very large decrease in the contact angle indicates a significant increase in the hydrophilicity of the surface, which is due to the high concentration of Fo and its alkaline effect. The hydrophilic surface can facilitate the adsorption of proteins, biological agents, and cells. This property is very desirable for bone scaffolds, as it improves the adhesion and proliferation of cells (such as osteoblast cells). Under biological conditions, the more hydrophilic surface allows for the rapid adsorption of calcium and phosphate ions from the body environment, which contributes to the mineralization process and bone formation (osteogenesis). Figure 3 shows a picture of the contact angle with a drop volume of 5 μm in the samples inserted.

**Figure 4:**
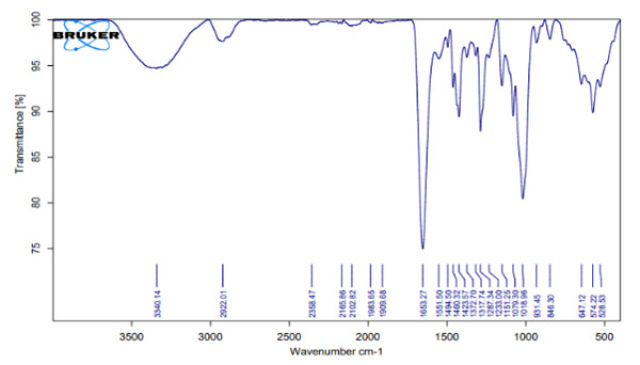
FTIR spectrum of the carboxyl methyl chitosan and PVP sample (sample S1)

FTIR: Fourier transform infrared spectroscopy; PVP: Polyvinylpyrrolidone

In the FTIR technique, functional groups and covalent bonds are identified based on the measurement of the absorption of IR radiation by the sample as a function of wavelength. In this way, the molecular components of their composition and structure can be determined. In the analysis of Figure 4 of sample S1, which contains carboxyl methyl chitosan and PVP, several key bands are observed in the infrared spectrum. The region between 3500-3200 cm^-1^ shows a broad band associated with the O-H stretching vibration of alcohols or carboxylic groups, as well as N-H of amines.

**Figure 5:**
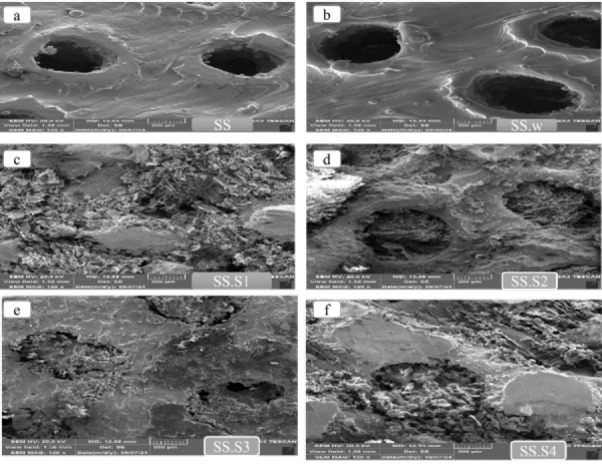
X-ray patterns of stainless-steel samples with different coatings: PBS removal, S1, S2, S3, and S4

 In the range of 2900-2800 cm^-1^, C-H stretching bands representing aliphatic bonds were detected. A distinct peak at 1650-1600 cm^-1^ was attributed to the C=O bond, indicating the carbonyl stretching vibrations of PVP and carboxylic groups. In addition, the presence of N-H bending bands from secondary amines was observed in the spectrum around 1550-1500 cm^-1^, and signals related to C-O bonds from alcohol and ether groups were detected at 1100-1000 cm^-1^. In sample S2, which contains carboxyl methyl chitosan and polyvinylpyrrolidone polymer and 5% of Fo, similar bands are seen as in sample S1, but the intensity of the bands, especially at 11650 and 1100 cm^-1^, has decreased due to the addition of 5% by weight of Fo. New peaks have appeared in the region of 900-600 cm^-1^, which are related to Si-O-Si vibrations in Fo. In sample S3, the intensity of C=O and N-H stretching bands has decreased compared to sample S2, indicating a greater interaction of Fo with the organic matrix. The Si-O-Si peaks in the 900-600 cm^-1^ region become clearer and show an increase in intensity. In sample S4, the peaks of organic groups O-H, N-H, and C=O have decreased again compared to sample S3, and Fo Si-O-Si has completely dominated and has a higher intensity. These changes indicate an increase in the chemical contribution of Fo in the structure of the compound. The decrease in the intensity of O-H and N-H bonds in samples S2, S3, and S4 indicates that Fo reduces the number of free hydroxyl groups. The C=O peak at 1650 is more intense in sample S1 and decreases with increasing Fo percentage in other samples, indicating the interaction of carboxyl with Fo. The Si-O-Si peaks in the 900-600 cm^-1^ region appear only in samples S2, S3, and S4 and become more intense with increasing Fo percentage. These changes indicate chemical interactions between Fo and the organic matrix, the effects of which are also observed in the SEM and XRD results. These peaks are characteristic of minerals and indicate the success of Fo addition. Bio-composite properties for bone scaffolds, S1 is suitable for initial biocompatibility and bioactivity properties. S2 and S3 have a good balance between organic and inorganic groups, which may provide better mechanical properties and biocompatibility for bone scaffold applications, and S4 has higher hardness due to the dominance of minerals. In the XRD analysis of 316 stainless steel, Figure 5, the SS sample can be identified with several characteristic peaks that are consistent with its crystallographic structure. The most prominent peaks for 316L stainless steel at 2θ angles, at 43.55°, 50.71°, and 74.70°, indicate a face-centered cubic (FCC) structure. The presence of sharp and distinct peaks in the SS sample indicates a well-ordered crystal structure, which contributes to the ability to deform without fracture, crack resistance, and load-bearing ability. In the SS. W sample, which is a stainless-steel sample after leaving the PBS solution, the peak intensity is reduced, and the peaks are broader compared to the image. The broadening of the peaks indicates a decrease in crystallite size or an increase in disorder in the crystal lattice. In the case of SS. S1, the metal sample is coated with PVP and carboxyl methyl chitosan polymers, which indicates that the polymer coating is well-attached to the stainless-steel metal surface. PVP is also generally considered amorphous, and carboxyl methyl chitosan is only slightly visible at 10 to 30 degrees 2θ.

 The XRD patterns of polyvinylpyrrolidone and carboxyl methyl chitosan result in a decrease in peak intensity compared to the SS sample, indicating an amorphous structure that enhances properties such as adhesion and flexibility. In the case of SS. S2, the XRD pattern of the metal sample coated with 5 wt% Fo polymers has a crystalline microstructure. By comparing the peak areas in SS. S3 and SS. S4, which are at 10 and 15 wt.%, the phase structure and crystallinity of the bone scaffold can be understood. As the percentage of Fo increases, the intensity of the peaks increases and the peaks become broader, indicating a crystalline phase (32).

**Figure 6:**
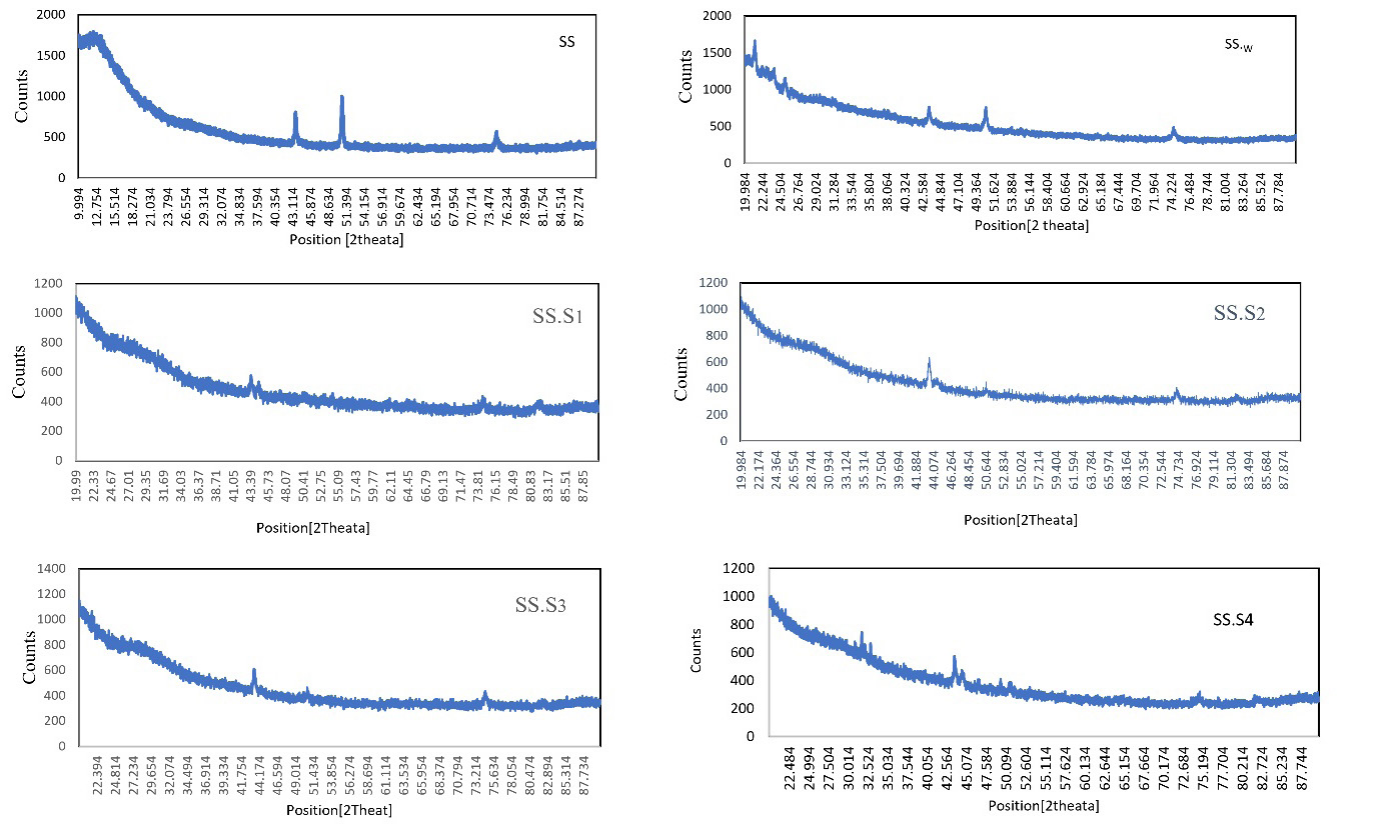
SEM images show stainless steel scaffolds from 3D printing: samples SS, SS.w, and various forsterite coatings (0%, 5%, 10%, and 15%)

 The morphology and geometry of the pore size are evaluated in Figure 6 (a-f). Sample SS represents the core of a 3D-printed stainless-steel scaffold. The surface of the sample is relatively smooth, and the lines resulting from the manufacturing process (3D printing) with well-defined circular pores indicate good precision in the 3D printing process, which are visible. The edges of the pores appear intact and well-defined. They are suitable for use in bone scaffolds where the pore architecture is critical for cellular attachment and tissue integrity and SEM images were taken after removal, showing that the surface of the sample had changed (as shown in Table 2 and Table 3). The accumulation of spherical particles around the pores and the surface of the sample is visible, which is due to mineral precipitation or weight loss in PBS solution. Compared to the SS sample, the edges are smoother, and their shape has undergone chemical changes. The SS sample shows the direct effect of mechanical loading without chemical changes. The SS. w sample has observed obvious changes of corrosion, surface dissolution, and accumulation of corrosion products or mineral deposits due to exposure to PBS. Sample SS. S1 has less distinct and irregular pores. The porous scaffold surface appears rough but lacks granular features, and the smooth surface indicates the absence of Fo particles. Sample SS. S2 has more distinct and rounded pores than sample SS. S1. This indicates that the freeze-drying process has better preserved microporosity. In this sample, some granular features are visible, indicating that Fo particles are incorporated into the scaffold. Adding 5 wt% Fo improves the texture and increases the mechanical and bioactive properties of the scaffold while maintaining reasonable porosity. In the sample SS. S3, the porosity is slightly reduced compared to the sample with 5 wt% Fo.

**Figure 7:**
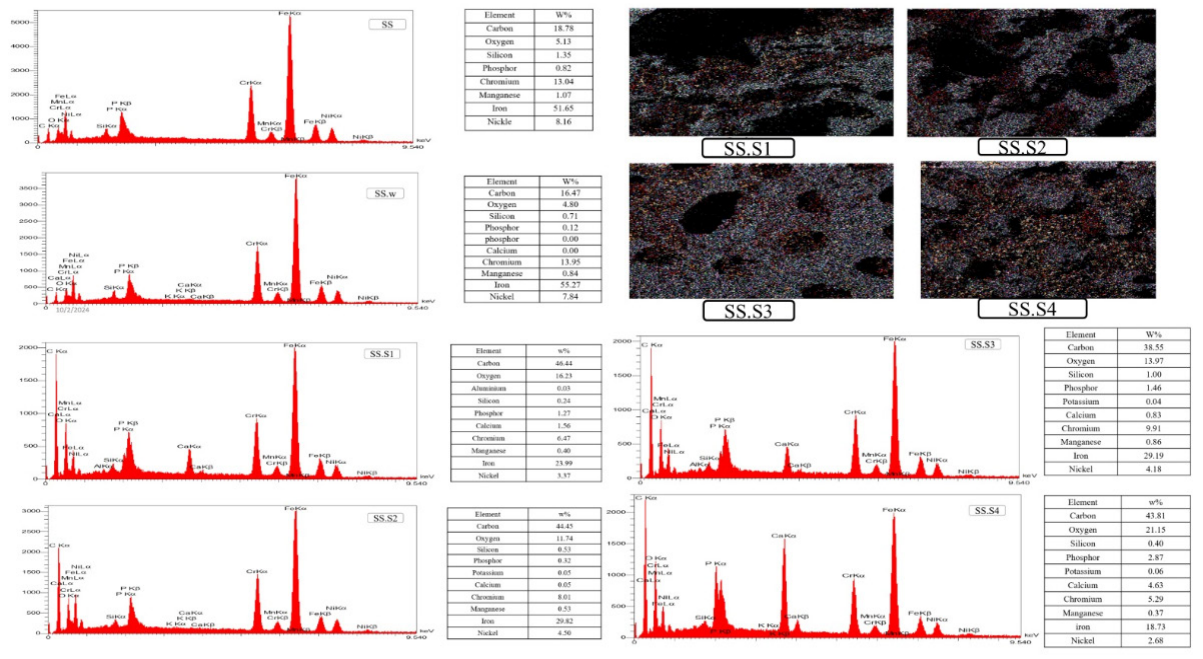
Energy dispersive X-ray spectroscopy (EDX) images of stainless steel samples SS and SS.w after PBS solution exposure, alongside scaffolds with varying forsterite content (S1, S2, S3w, S4w)

 With increasing Fo amount, the roughness of the porous scaffold increases, which is due to the cell attachment in biomedical applications. In the sample SS. S4, the pores are further reduced and many of them are blocked or filled, and the scaffold surface is heavily filled with particles, indicating that it is 15% Fo, which significantly affects the surface morphology. EDX analysis of scaffolds made of stainless steel and other compounds has been investigated. The characteristics of each sample and the results of EDX spectrum analysis are given in Figure 7. Sample SS contains the main elements of stainless steel, which are Fe, chromium (Cr), and nickel (Ni). 

The peaks of these elements are observed with high intensity in the EDX diagram. The intensity of the Cr peak indicates that this sample has a suitable chromium protective layer, which ensures corrosion resistance. Also, the Fe and Ni peaks remain without any special changes. Sample SS. w in this sample, the main elements Fe, Cr, and Ni are also observed, but significant changes are seen in the intensity of the Cr and Fe peaks. The decrease in the Cr peak indicates the decrease in the chromium protective layer, which can be due to the weight loss. The relative increase in the Fe peak also indicates that the metal base surface is more exposed to the PBS environment. The possibility of the presence of an oxygen (O) peak also indicates the formation of surface oxides. This sample has lower corrosion resistance due to surface changes and has been more affected by the PBS environment. The SS sample has a stronger protective layer than the SS.w sample. This is evident from the intensity of the Cr peak in the graph. In the SS. w sample, the formation of oxides and more chemical changes were observed, which reduced its corrosion resistance. The relative increase in the Fe peak in the SS.w sample indicates the removal of the chromium layer and the greater exposure of the metal base surface. 

## Discussion

The SS sample showed better resistance to PBS solution, and few changes were observed in its chemical structure. In contrast, the SS.w sample was more vulnerable due to the reduction of the chromium layer and the formation of oxides and was more affected by the corrosive environment of PBS. In the SS. S1 and SS. S2 samples, the long peaks of iron, chromium, and nickel indicate the three main components of stainless steel, and the small peaks corresponding to C and O atoms are identified, which are attributed to carboxyl methyl chitosan and PVP. In the SS. S2 sample, weak peaks for magnesium and silicon are shown in the analysis due to the addition of 5 wt% Fo (Mg_2_SiO_4_). In the sample SS. S3, it is observed that the intensity of Mg and Si peaks has increased compared to sample SS. S2. Stainless steel elements continue to dominate with increasing Fo penetration. In the sample SS. S4, the peaks of magnesium and silicon have grown strongly. 

**Figure 8:**
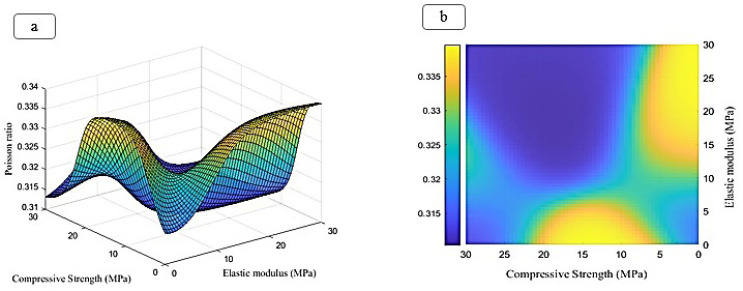
Prediction made by the ANN to predict the Poisson ratio in this study, (a) front view, (b) side view

**Figure 9:**
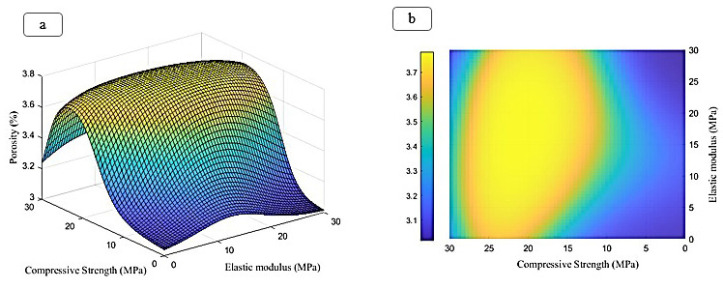
Prediction made by the ANN to predict the porosity percentage of the tested material in this study, (a) front view, (b) side view

**Figure 10:**
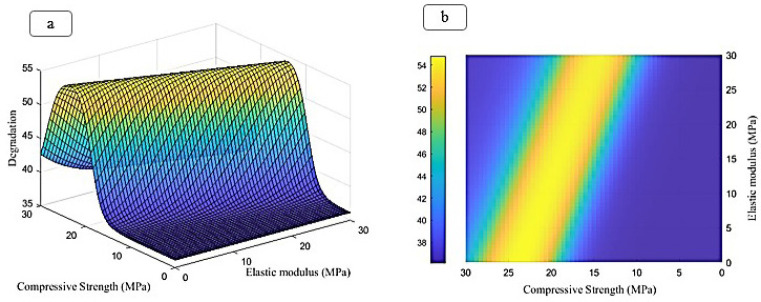
Prediction made by the ANN to predict the degeneration in this study, (a) front view, (b) side view

**Figure 11:**
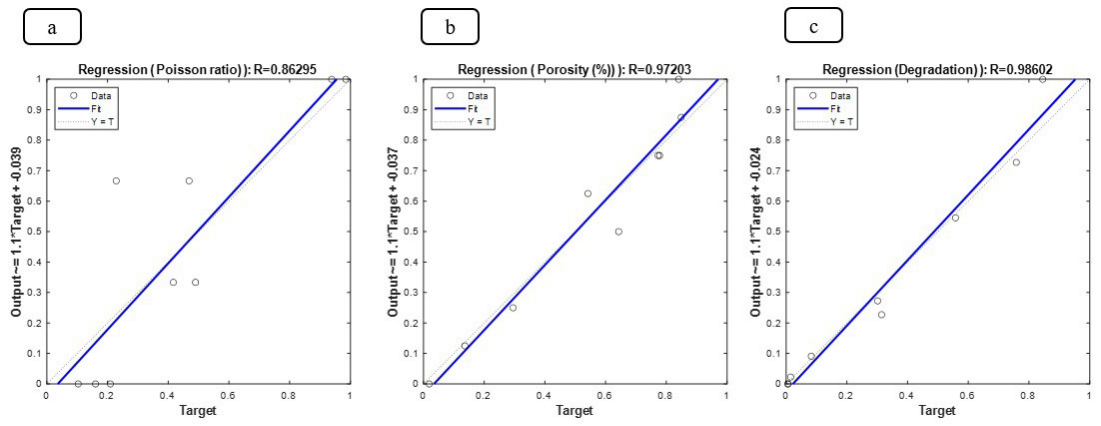
Linear regression plots to investigate the error of the ANN formed in this study (a) Poisson's ratio, (b) porosity percentage, and (c) degradation rate

The ratio of peaks of iron, chromium, and nickel decreases relatively. In general, all samples have the basic composition of stainless steel, which is modified by the addition of other compounds. At an increasing weight percentage of Fo from 5% to 15%, the peaks of magnesium and silicon are continuously strengthened. The presence of carboxyl methyl chitosan and PVP in the EDX spectrum is less due to their combination with lighter elements such as C and O, since these elements are difficult to detect by EDX. Peaks of iron, chromium, and nickel are present in all samples, but their relative intensity decreases with increasing Fo percentage. Increasing the weight percentage of Fo has a direct effect on the presence and intensity of the magnesium and silicon peaks in the spectrum. However, stainless steel is the basic composition in all samples. Adding compounds, such as Fo and carboxyl methyl chitosan, only creates new features in the structure. To predict the change in Poisson’s ratio, porosity percentage, and degeneration with increasing elastic modulus and compressive strength, an FFANN is formed according to Table 1. The indicative behavior, including changes in Poisson’s ratio, porosity percentage, and degeneration with elastic modulus in the range of 0 to and compressive strength in the range of 0 to 60, is predicted and investigated. Figure 8 (a-b) shows the results predicted by the neural network for the rate of change in Poisson’s ratio. It can be concluded from the conditions that increasing the elastic modulus causes the growth of Poisson’s ratio. Also, changes in compressive strength cause the growth of Poisson’s ratio in a range and then decrease. Figure 9 (a-b) illustrates the neural network’s predictions regarding the rate of change in porosity percentage. The analysis indicates that increasing the elastic modulus does not significantly impact porosity; initially, it increases slightly before decreasing. An increase in compressive strength leads to a rise in porosity percentage, which subsequently declines after reaching 20 MPa. Figure 10 (a-b) shows the neural network’s predictions for the rate of change in degeneration. The degeneration pattern closely resembles that of porosity percentage, with increasing compressive strength initially promoting degeneration; however, this trend reverses after 20 MPa. Figure 11 (a-c) shows the results of linear regression analysis concerning the inputs and outputs. As anticipated, the artificial neural network demonstrated a high level of accuracy in predicting Poisson’s ratio, porosity percentage, and degeneration, achieving an error margin of less than 1% compared to the targets outlined in Table 1.

As expected, the ANN was able to predict Poisson’s ratio, porosity percentage, and degeneration with very high accuracy and an error of less than 1 percent compared to the targets considered in Table 1. By creating the elastic modulus and compressive strength, the parameters of Poisson’s ratio, porosity percentage, and shrinkage can be changed. Poisson’s ratio is at its highest when the elastic modulus is at its highest and the compressive strength is at its lowest. It is also worth noting that Poisson’s ratio is directly related to the elastic modulus. The porosity percentage and shrinkage are at their highest when the compressive strength is within 20 MPa, and the elastic modulus has no effect on the result. It is also worth noting that the porosity percentage and shrinkage had a direct relationship with the compressive strength. Many studies (33-35) have shown the increasing use of advanced computational methods, including machine learning and predictive modeling, in materials science and engineering. These approaches are particularly applicable to your research, which utilizes ANNs to forecast the mechanical and biological properties of your multicomponent bone scaffold. The referenced studies explore innovative applications of nanotechnology and advanced materials in biomedical fields. Recent research has shown innovative materials and techniques for medical and structural applications. N,O-carboxymethyl chitosan has been developed as an antibacterial wound dressing, while electroconductive scaffolds are being utilized for bone cancer therapy (36-40). Advancements in 3D Bioprinting led to the creation of a prototype mitral heart valve using sustainable polymers. Additionally, optimized alginate scaffolds show promise for alveolar bone regeneration (41-45). Studies on carbon nanotubes reveal their significant impact on the thermal properties and mechanical performance of composite materials, enhancing their applicability in various engineering fields (46-49).

Recent advancements in various fields show significant innovations (50-54). In the realm of materials science, 3D-printed polymer-based bone scaffolds are emerging as promising solutions for biomedical applications (55-59). Meanwhile, research into allergens, such as Der p 22, sheds light on their immunogenicity, particularly in asthmatic children. Machine learning techniques are being utilized to predict high-temperature ablation resistance in ceramic coatings, further enhancing material performance (60). Additionally, the development of bioinspired flexible composites and nanomaterials shows the potential for improved personal protective equipment and cancer treatment strategies.

ANN: Artificial neural networks

PBS: Phosphate-buffered saline

**Table 1:** Mechanical properties and ANN predictions for porosity and poisson ratio of tested samples

**Porosity** **(%)**	**Poisson ratio**	**Compressive strength** **(MPa)**	**Elastic modulus** **(MPa)**	**Sample**
**60**	0.31	3.2	42	SS
**58**	0.31	3.6	48	SS.S_1_
**57**	0.33	3.7	52	SS.S_2_
**55**	0.32	3.8	58	SS.S_3_
**52**	0.34	3.4	41	SS.S_4_
**48**	0.31	3.6	38	S_1_
**46**	0.33	3.5	36.5	S_2_
**41**	0.32	3.1	36	S_3_
**40**	0.34	3.0	36	S_4_

ANN: Artificial neural networks

ANN: Artificial neural networks

ANN: Artificial neural networks

**Table 2:** Ion release test analysis (ICP-AES) for calcium levels in various samples and controls

Sample										
S4	S3	S2	S1	SS. S4	SS. S3	SS. S2	SS. S1	SS. w	Control sample (PBS)	
388.696	86.2108	111.064	39.9399	239.820	87.3875	21.9914	58.8468	17.2089	0.529511	Ca ppm

ANN: Artificial neural networks

**Table 3:** Biological properties of samples: Degradation, ICP-AES, Bioactivity, and pH measurements

**pH**	**Bioactivity**	**ICP-AES**	**Degradation**	**Sample**
**6.6**	0.22	17.20	0.06	SS
**6.52**	0.21	58.84	0.09	SS. S_1_
**6.6**	0.24	21.99	0.08	SS. S_2_
**7.1**	0.34	87.38	0.06	SS. S_3_
**7.1**	0.28	239.82	0.06	SS. S_4_
**6**	0.32	39.93	0.18	S_1_
**6**	0.35	111.06	0.17	S_2_
**6**	0.36	86.21	0.12	S_3_
**6**	0.37	388.69	0.34	S_4_

## Conclusion

In this study, a three-component metal-polymer-ceramic scaffold was fabricated using a metal 3D printer and freeze-dried coating. In this regard, nine samples were obtained, and then analyses and tests were used to analyze and identify the bone scaffold. In the ion release test, adding growth increases the release of calcium ions and changes the pH to alkaline, which is beneficial for the movement of bone mineralization. Also, the results of the wettability test showed that increasing the amount of Fo improved the hydrophilicity of the sample surface. For example, with a 15 wt% speed, the minimum contact angle reached 4.45 degrees, which can help improve cells and biological processes. These changes in properties can have an important impact on the performance of bone scaffolds, especially in the field of cell adhesion and proliferation. In FTIR samples, various functional groups such as O-H, C-H, C=O, and N-H were identified. With the increasing Fo percentage, the intensity of O-H and N-H bonds decreased, and the Si-O-Si bands of Fo particles increased. Samples with a high percentage of industry (S4) have better mechanical properties and biocompatibility. XRD results showed a new crystal structure and improvement of the crystal phase with increasing Fo. SEM images showed that with increasing Fo, the pore size decreased, and the roughness increased. In EDX analysis, with increasing Fo, the Mg and Si peaks increased further. In this study, an ANN is used to predict Poisson’s ratio, porosity percentage, and degeneration based on elastic modulus and compressive strength. The results showed that with increasing elastic modulus, Poisson’s ratio increases, and porosity percentage is affected by compressive strength, first, and then. Also, the degeneration behaves similarly to the porosity percentage. These results make it necessary to carefully select the loading conditions to optimize the performance of scaffolds in clinical applications. Neural network was introduced as a tool for designing bone scaffolds.

## Declaration

We have not used any AI tools or technologies to prepare this manuscript.
